# Endovascular repair of aortic arch graft pseudoaneurysm using a duct occluder device with onlay fusion guidance

**DOI:** 10.1016/j.jvscit.2022.09.006

**Published:** 2022-09-20

**Authors:** Guilherme B. Barbosa Lima, Laura Ocasio, Emanuel R. Tenorio, Marina Dias-Neto, Thanila A. Macedo, Gustavo S. Oderich

**Affiliations:** a Department of Cardiothoracic and Vascular Surgery, Advanced Aortic Research Program, The University of Texas Health Science Center at Houston, McGovern Medical School, Houston, TX; b Department of Diagnostic and Interventional Imaging, The University of Texas Health Science Center at Houston, McGovern Medical School, Houston, TX

**Keywords:** Aortic arch, Ductal occluder device, Endovascular aortic arch repair, Onlay fusion, Percutaneous aortic arch repair, Pseudoaneurysm

Aortic arch pseudoaneurysms can be caused by previous surgery, infection, or trauma. In select patients with small tears and no evidence of infection, local repair can be performed using an open or endovascular technique.^1^ Open surgical repeat surgery has increased risks and endovascular repair has been especially appealing for the elderly, higher risk patients, and patients with multiple prior procedures.^2^ We have reported the use of a ductal occluder device in a patient with a large aortic arch pseudoaneurysm.^3^ Our patient has provided written informed consent for the report of his case details and imaging studies.

A 71-year-old man had presented with a contained ruptured pseudoaneurysm in the midportion of the aortic arch graft. His medical history included a DeBakey type 1 dissection that had requiring ascending aortic repair 21 years earlier, aortic valve replacement, total aortic arch repair, and repeat aortic root and arch replacement. Dynamic computed tomography angiography and echocardiography confirmed the presence of a perivalvular leak without communication with the pseudoaneurysm and a diminutive tear in the inner curvature of the surgical graft without evidence of infection.

Initially, the patient had undergone a failed endovascular repair by the interventional cardiology team without successful catheterization of the tear. Subsequently, analysis of the dynamic computed tomography angiography (CTA) accurately located the graft defect, allowing for the creation of onlay fusion for intraoperative guidance. The procedure was performed in a hybrid room (Video) using general anesthesia and a percutaneous femoral approach with an 8.5F, 90-cm, Aptus TourGuide (Oscor Inc, Palm Harbor, FL). After onlay fusion calibration, the defect was catheterized using a VS1 catheter and glidewire, which were exchanged for a 4F Kumpe catheter and Rosen wire (Cook Medical Inc, Bloomington, IN). A 5F, 110-cm flexor sheath (Cook Medical Inc) was advanced, followed by placement of a 6-mm Amplatzer ductal occluder II (Abbot Medical, Plymouth, MN). Cone-beam computed tomography confirmed the position of the device, and it was released from the delivery system.

He was discharged on the fifth postoperative day without complications. The CTA obtained before discharge revealed a smaller, but persistent, endoleak. A repeat CTA at 6 weeks revealed endoleak resolution and a significantly smaller pseudoaneurysm. The patient had experienced a hemorrhagic stroke at 42 days postoperatively due to anticoagulation therapy for the prosthetic aortic valve. However, he had recovered without sequelae. He had had no other complications at the 6-month follow-up. The present case has highlighted the importance of imaging guidance to locatsmall lesions in difficult projections.
